# Cell Lines for Honey Bee Virus Research

**DOI:** 10.3390/v12020236

**Published:** 2020-02-20

**Authors:** Ya Guo, Cynthia L. Goodman, David W. Stanley, Bryony C. Bonning

**Affiliations:** 1 Department of Entomology and Nematology, University of Florida, Gainesville, FL 32611, USA; ya.guo@ufl.edu; 2 Biological Control of Insects Research Laboratory, USDA/Agricultural Research Service, Columbia, MO 65203, USA; cindy.goodman@usda.gov (C.L.G.); david.stanley@usda.gov (D.W.S.)

**Keywords:** honey bee virus, Hymenoptera, insect cell culture, cell lines, *Apis mellifera*, deformed wing virus

## Abstract

With ongoing colony losses driven in part by the Varroa mite and the associated exacerbation of the virus load, there is an urgent need to protect honey bees (*Apis mellifera*) from fatal levels of virus infection and from the non-target effects of insecticides used in agricultural settings. A continuously replicating cell line derived from the honey bee would provide a valuable tool for the study of molecular mechanisms of virus–host interaction, for the screening of antiviral agents for potential use within the hive, and for the assessment of the risk of current and candidate insecticides to the honey bee. However, the establishment of a continuously replicating honey bee cell line has proved challenging. Here, we provide an overview of attempts to establish primary and continuously replicating hymenopteran cell lines, methods (including recent results) of establishing honey bee cell lines, challenges associated with the presence of latent viruses (especially *Deformed wing virus*) in established cell lines and methods to establish virus-free cell lines. We also describe the potential use of honey bee cell lines in conjunction with infectious clones of honey bee viruses for examination of fundamental virology.

## 1. Introduction

About one third of all agricultural crops are dependent on the honey bee (*Apis mellifera*) for pollination, reflecting the importance of the honey bee to agricultural production. However, honey bee colonies in the northern hemisphere have been in decline [1,2,3,4,5]. With an estimated 59% total loss of colonies between 1947 and 2005 [1], and an average 40% annual loss of colonies from 2010 to 2016 [6,7,8,9,10], these declines are of ongoing concern [11]. While the causes of honey bee colony decline are complex [2], the ectoparasitic mite, *Varroa destructor*, represents a major threat to honey bee health [12,13]. In addition to weakening honey bees by feeding on body fat [14], the Varroa mite also vectors honey bee viruses [15,16,17,18,19,20], with the spread of the Varroa mite resulting in dominance of a more pathogenic *Deformed wing virus* (DWV) strain [16,21]. At least 24 honey bee-associated viruses have been reported [22], including seven viruses that are widespread. These are *Acute bee paralysis virus* (ABPV), DWV, *Sacbrood virus* (SBV), *Black queen cell virus* (BQCV), *Israeli acute paralysis virus* (IAPV), *Chronic bee paralysis virus* (CBPV), and *Kashmir bee virus* (KBV) [22,23]. 

Insect-derived cell lines provide valuable tools for the study of insect viruses under controlled conditions, with their genetic uniformity providing for more consistent results. Cell lines may allow for the study of suborganismal processes that may not be tractable using the host organism. Insect cell lines can also be used for the screening of insecticides or biocontrol compounds against pests, or for the assessment of potential risk to nontarget organisms such as the honey bee [24]. Approximately 1000 insect-derived cell lines have been established according to the ExPASy Cellosaurus database, with >80% derived from Diptera and Lepidoptera [25]. However, relatively few cell lines are derived from Hymenoptera. 

A honey bee-derived cell line would provide a valuable tool for the study of virus–insect and virus–virus interactions. In this review, we provide a summary of the establishment of primary cultures and continuously replicating hymenopteran cell lines, virus studies using the *Apis mellifera*-derived AmE-711 cell line, methods for the establishment of virus-free cell lines, and potential applications of these cell lines in insect virology. A honey bee cell line would provide a powerful research platform for the increased understanding of honey bee virology. 

## 2. Establishment of Hymenopteran Cell Lines

### 2.1. Primary Cell Lines

A primary cell line is a cell line derived from specific insect tissues or organs, cultured on an artificial medium and maintained for a limited time. Primary cell cultures have been established from three hymenopteran species, including an ant (*Pseudomyrmex triplarinus*), a parasitic wasp (*Mormoniella vitripennis*), and the honey bee (*A. mellifera*) (Table 1) [26,27,28,29]. The longevity of these primary cell cultures was highly variable. Primary cell cultures derived from ant venom gland cells were maintained for up to 12 months, while honey bee primary cell cultures were viable from days to months [26,27,28]. Most of the early primary cell cultures from the honey bee were derived from neural tissues (Table 1) [30,31,32,33,34,35,36,37,38,39,40]. An early primary neuron-derived culture, dissociated by mechanical treatment and prepared from specific regions of the pupal brain, survived for only three weeks [31]. Importantly, the cultured neurons showed surface properties and a transmitter phenotype similar to those of their in vivo counterparts [41], indicating the potential for primary cell cultures in the study of cell biology. Additional honey bee primary cell lines were established from eggs [42,43,44,45], guts [41,46] and larval or pupal tissues (Table 1) [27,35,41,47,48,49]. 

Similar procedures were used for the generation of these primary cell cultures, as follows [28]. (1) Bees or tissues were surface sterilized using a sterilization buffer containing ethanol, hypochlorite or H_2_O_2_, and rinsed several times. (2) The tissue was gently homogenized or torn apart in a specific growth medium (e.g., L-15 cell culture medium, originally established for mammalian cell culture) with several types of antibiotics (e.g., gentamycin, penicillin, streptomycin), and an antimycotic (e.g., amphotericin B). Mechanical methods were typically used for the establishment of honey bee primary cell lines, as enzymatic dissociation of tissues resulted in limited numbers of isolated cells and contamination [41]. (3) The homogenate was transferred to an incubator with the medium replaced at intervals, until the expected morphology of the cells was observed. Primary cell types may be adherent or non-adherent (floating). (4) The identity of the cells was confirmed by polymerase chain reaction (PCR) amplification of a specific gene sequence from DNA extracted from cultured cells, and sequencing of the PCR product. The target genes included *actin* and *laminin* for confirmation of honey bee cell lines [43,47]. Mitochondrial *cytochrome c oxidase subunit I* (*COI*) is also commonly employed for this purpose. 

The cell culture medium used significantly influenced cell growth rates, suggesting that specific nutrients are required for maintenance of honey bee cells. The media that support the growth of cell lines derived from other insects were mostly insufficient for the maintenance of honey bee-derived cells. The evaluation of different media for cell growth was required, with cells growing extremely slowly in an unsuitable environment. For example, primary cells of *A. mellifera* were reported to show attachment and growth in WH2, a medium modified from HH-70 psyllid culture medium, while they grew slowly in two commercial media, Sf-900™III SFM and EX-CELL 405 [47]. Chan et al. (2010) transduced bee cells using lentivirus, illustrating the use of molecular manipulations for developing immortal cell lines. In this study, insect cell culture media (Grace’s and Schneider’s) and mammalian cell culture media were compared with the former, resulting in higher viability. Cryopreservation of bee cells was also demonstrated for short-term storage. Two media were recommended (BM3 and L-15) by Genersch et al. [28] for the isolation and cultivation of neuronal cells from pupae or adults, and gut cells from pupae. Ju and Ghil used L-15 medium-based honey bee cell (LHB) growth medium and Schneider’s insect medium-based honey bee cell (SHB) growth medium, with more cells produced in the LHB medium than in SHB medium after six passages. The doubling time in LHB medium was only about eight days [43]. Clearly, identification of a suitable cell culture medium is critical for the maintenance of primary cell cultures.

### 2.2. Continuous Cell Lines Derived from Hymenoptera

A continuous cell line is often comprised of multiple cell types and can be passaged in culture for many generations or indefinitely [50]. In the Class Insecta, many well-characterized cell lines derived from Lepidoptera and Diptera have been described [25,51,52]. However, relatively few continuous insect cell lines from Hymenoptera have been reported (Table 2). These include cell lines derived from *Neodiprion lecontei* (Diprionidae) [53], *Trichogramma pretiosum* (Trichogrammatidae) [54], *T. confusum*, *T. exiguum* [55] and *Hyposoter didymator* (Ichneumonidae) [56] (Table 2). To our knowledge, replication of honey bee viruses in these cell lines has not been tested. 

The establishment of a continuous cell line from the honey bee has proven difficult, with only two continuous cell lines reported (Table 2). Bergem et al. investigated the long-term maintenance of honey bee cells by generating cell cultures derived from different honey bee tissues and testing several culture media. Cell cultures were initiated from a specific stage of the honey bee embryo, the pre-gastrula stage, and cells remained mitotically active for more than three months [44], suggesting that honey bee embryos at this specific stage provide good starting material for long-term cultivation. Kitagishi Y et al. engineered *A. mellifera* cells derived from honey bee embryos using the human c-myc proto-oncogene for their long-term cultivation [57]. The cell line, designated as MYN9, was successfully cultured for more than 100 generations over a period of more than eight months, suggesting that the human c-myc proto-oncogene was efficient for immortalization of honey bee cells. Honey bee marker genes and c-myc were detectable by PCR. However, the honey bee virus, *Deformed wing virus* (DWV), was also detected in the MYN9 cell line. While MYN9 was a honey bee-derived cell line, whether expression of c-myc in the cells affected endogenous gene expression or not is unknown.

A honey bee cell line derived from embryonic tissues, named AmE-711 (*Apis mellifera* cell line from Embryonic tissues, established on 7/2011), was reported by Goblirsch M. et al. [58,59] Similarly, mid to late stage honey bee eggs were used as the initial material for the establishment of primary cultures, as undifferentiated embryonic cells are continuously dividing. The AmE-711 cell line was isolated from one of multiple primary cell lines. Several challenges were encountered during the establishment of the AmE-711 cell line: (1) It took time for the honey bee cells to adapt to the culture as most of the primary cultures required three months to reach confluence [58]; (2) Only one out of ~100 subsequent cell passages from primary cell cultures continued to replicate [58]; (3) The length of time used for enzymatic treatment significantly influenced cell fate. Incubation with trypsin for more than 10 min led to failure of cell re-attachment or cell injury [58]. 

The AmE-711 cell line contained bipolar and multipolar fibroblastic cells, elongated in shape with an adherent growth phenotype. Most cells had a diploid karyotype, similar to honey bee cells in nature. Most importantly, the cell line was continuous, as it was maintained long term and passaged at least 18 times, with a minimum of 43 generations [58,60]. However, the AmE-711 cell line proved difficult to maintain and crashed in 2015, possibly due to virus infection (see Section 3 below). Fortunately, this cell line has since been recovered and adapted to a commercially available medium (Dr. Michael Goblirsch, USDA, ARS; personal communication). 

### 2.3. A Systematic Iterative Protocol to Establish Tissue-Derived Insect Cell Lines from Honey Bees and Other Challenging Insect Species: Recent Results from BCIRL 

Hundreds of insect cell lines have been established since the first ones were produced in the late 1950s and 1960s [61,62]. Some of these lines are in routine use in industry, university, and government laboratories. The Biological Control of Insects Research Laboratory (BCIRL) has a history of establishing cell lines [63,64,65,66,67,68], generally using a standard protocol. This protocol has a core set of steps systematically repeated with observation-based changes in media components that ultimately lead to established, functional cell lines. A suitable medium based on experience and the literature is selected for the first cell line initiation. In later iterations, cell lines are initiated with other media, and sometimes with new media created by mixing known media or by adding media supplements. This iterative process generally leads to the establishment of permanent cell lines, useful in several research and development programs [65,67,68]. 

In recent years we have been working to establish cell lines from honey bees at BCIRL. The establishment of cell lines derived from honey bees has proven to be very difficult, similar to the situation for a large group of insects from a variety of orders. It is not clear why cell lines are routinely established from some orders of insects, such as Lepidoptera, but not others. Such differences in cell line establishment may relate to fundamental cellular biology. We plan to investigate the point in detail by tracing gene expression patterns during the establishment process, using cell lines from lepidopterans and coleopterans that are routinely established, and from recalcitrant species, similar to work in *Drosophila melanogaster* cell lines [69]. 

All bee stages were used for culture initiations, including adult workers and queens and specific tissues within the bees. We initiated lines with the midgut, nervous system (ventral nerve cord, brain, or both), aorta, fat body, ovaries, spermatheca, a combination of testes/fat body, muscle, Malpighian tubules, venom sack, and ground pupal heads.

**Figure 1:**
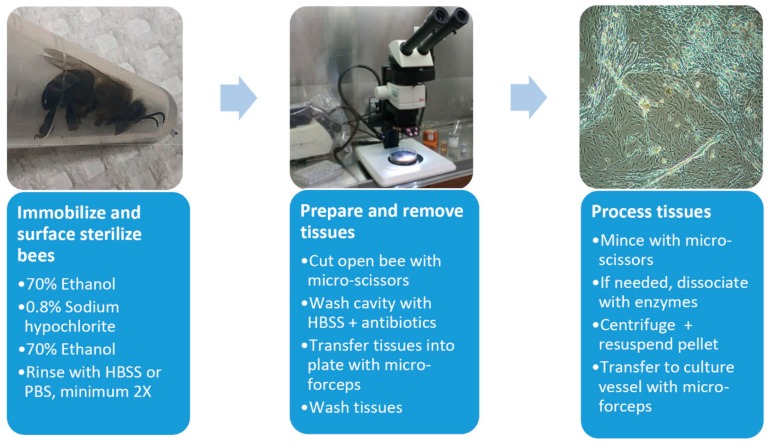
Flow chart for establishment of honey bee-derived cell lines. HBSS, Hanks balanced salt solution. See text for further details.

Cell culture initiations were performed in biosafety hoods with surface sterilized dissecting implements (Figure 1). Before dissection, the bees were immobilized in 70% ethanol (1 min) and surface sterilized in a series of treatments, 0.8% sodium hypochlorite (2–3 min), 70% ethanol (3–5 min) and rinsed 2–7 times in Hanks balanced salt solution (HBSS) or calcium, magnesium free–phosphate buffered saline (CMF-PBS). Bees were pinned dorsal-side up and an incision was made through the thorax and abdomen. The opening was flushed with HBSS containing antibiotics (0.1 mg/mL gentamycin, 0.5 μg/mL amphotericin B and/or 50–200 U/mL penicillin, 0.05–0.2 mg/mL streptomycin, Millipore Sigma), and selected tissues were removed with sterilized micro-forceps, washed three times in HBSS, and collected in wells of a standard 24-well tissue culture plate. Tissues were minced with sterilized micro-scissors, centrifuged if needed (800× *g*, 5 min, 4 °C), then transferred into either tissue culture plates (12-, 24-, or 48-well) or flasks (T_12.5_, T_25_) using cell culture media augmented with selected antibiotics (50–200 U/mL penicillin, 0.05–0.2 mg/mL streptomycin). In some initiations, 0.5 mL of an enzyme mixture (1 mg/mL collagenase/dispase, 0.05 mg/mL trypsin, Millipore Sigma) was added to dissociate the tissues. Enzyme-inoculated cultures were incubated at room temperature for 1h with gentle shaking. The dissociated tissues were centrifuged (800× *g*, 5 min, 4 °C), and transferred to culture containers as described. 

For smaller bee larvae (<4 mm), we minced the whole bodies immediately after sterilization. The eggs were collected into 1.5 mL microfuge tubes containing medium and gently agitated so that they remained in suspension. They were sterilized and washed as above, then either minced with micro-scissors or ground with a pestle. Cell cultures were maintained at 28 or 33 °C and observed daily. Insect cell lines are usually maintained at 28 °C [61,63,64,65,66,67,68]. We chose 33 °C as a comparison temperature, because the honey bee brood nest temperature is normally maintained at 33–36 °C for larval and pupal development [70]. The cultures were fed every 4 to 14 days (either by adding medium or replacing half, with these final concentrations of antibiotics: 50 U/mL penicillin, 0.05 mg/mL streptomycin). 

Over 600 honey bee cell cultures have been initiated using various combinations of tissues, media and media additives (Table 3). An iterative process was conducted for developing cell lines, i.e., we observed each culture initiated before deciding on the next media formulation to test.

The influence of the media + FBS on overall cell health was evaluated by visual inspection before testing different combinations of basal media or comparing the effects of supplements (nutritional or hormonal) added to the media. For example, we initiated cell lines with CLG#2, a combination of an insect cell culture medium (EX-CELL 420) and a mammalian cell culture medium (L-15), used to establish lepidopteran and hemipteran cell lines [67,68]). The HZ media mixture series began with the observation that CLG#2 produced healthy bee cell cultures. This was followed by testing different ratios of the same basal media (HZ#1), which did not lead to cell replication. Next came the replacement of one mammalian cell culture medium for another (RPMI-1640 for L-15, HZ#2), which generated healthy cells similar to CLG#2. The next two media combinations (HZ#3 and #4) were detrimental to cell viability. Similar iterations continued, each with a variety of media combinations ± additives. In this process, we found that royal jelly positively influenced bee cell health, although the underlying mechanisms for this improvement are not understood.

We paid particular attention to potential sources of contamination during cell line establishment. Fungal contamination may occur in bee cell culture initiations, although in most cases, this is controllable through surface sterilization and tissue washing as described. For tissues other than neonates and eggs, a fungicide at low levels (e.g., 0.5 µg/mL amphotericin B) was initially incorporated into culture media to minimize contamination. Another potential source of contamination was the accidental inclusion of small hive beetle (*Aethina tumida*) tissues within primary cultures. Adult beetles laid eggs in capped brood cells, as well as throughout the hive, and these eggs could have been mistaken for honey bee eggs [72]. *A. mellifera* only lay one egg per cell, while *A. tumida* can lay 10–30 eggs per cell, with the beetle eggs about 2/3 the size of honey bee eggs. *A. tumida* larvae are smaller than honey bee larvae, but they are more active, especially during their wandering stage (https://beeaware.org.au/archive-pest/small-hive-beetle/#ad-image-0 [accessed 12/9/2019]). We took care to ensure that only honey bee eggs and larvae were collected when initiating primary tissue cultures.

**Figure 2:**
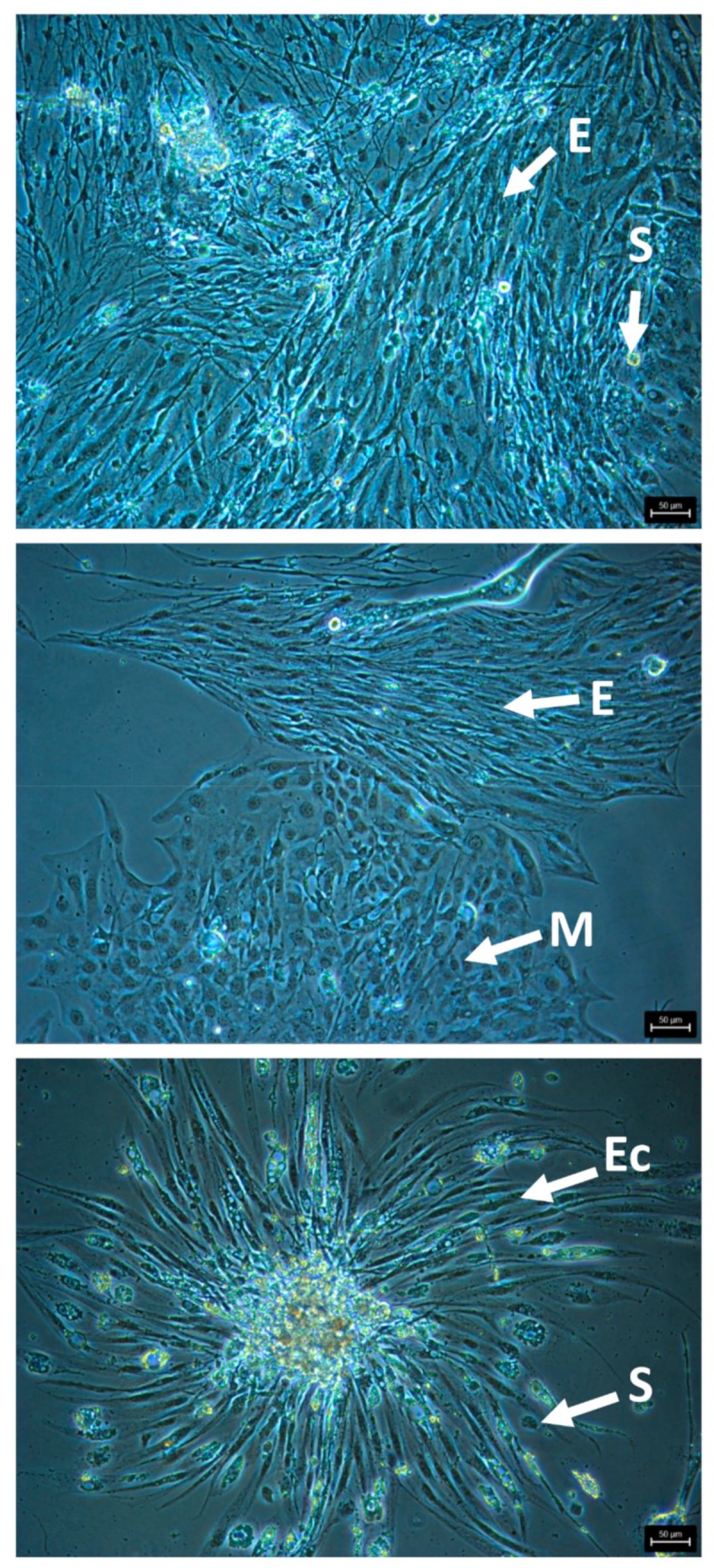
Representative images of attached, healthy cells from honey bee egg cell cultures in CLG#2 + FBS, passaged once, showing morphologically distinct cell types. E, elongated cells; S, spherical cells; M, multi-sided cells; Ec, elongated cells growing out of a cell clump. Bars, 50 µm.

The most promising and cleanest cultures were generated from eggs. Promising cultures consist of viable appearing, attached cells, with a clear cytoplasm, no vacuoles or darkened areas, and distinct cell membranes that are actively replicating (Figure 2). Cultures in CLG#2 + FBS +/- royal jelly led to the healthiest and longest enduring egg cell cultures. We passaged eight egg cultures at least once using 0.5% trypsin (3–5 min), and maintained the most promising cultures at 33 ^o^C. HZ#2 medium also produced viable/replicating cell cultures, although none were passaged. These latter cultures have a distinct major cell type, different from cells in the CLG#2 medium. Short-term egg cell cultures (one to five months) were initiated with TNM-FH and Schneider’s + FBS. 

Other short-term honey bee cell cultures (<1 month) that exhibit tissue and cell attachment, but no or minimal cell replication, include those initiated from the worker nervous system (in HB-1 or TNM-FH + FBS), larval/worker/pupal midgut (in HB-1 or CLG#2 + FBS + YE), ground pupal whole head (in CLG#2 + FBS), pupal nervous system (in HB-1), queen ovaries (using most basal media + FBS + other supplements, and WH5 or Kimura’s or HZ#1), queen midgut (in CLG#2 or TNM-FH or Kimura’s +/- other supplements) and queen/worker Malpighian tubules (in HZ#2 or Kimura’s + FBS). Some ovarian cell cultures exhibited cell networking with contractions. Based on these responses to different media configurations, we propose that each tissue has its own nutrient/medium requirements, which may reflect the in vivo situation. The tissues with the least stringent requirements for generating short-term cultures, aside from egg cell cultures, are those from queen ovarian tissues. This is generally true for cell lines initiated from ovaries and eggs, presumably due to the presence of undifferentiated cells. Clearly, more work is necessary to optimize the medium needed for each tissue isolate.

## 3. Cell Lines for Honey Bee Virus Studies 

Insect viruses typically infect cells derived from the host insect or from closely related species, with a few exceptions (e.g., *Cricket paralysis virus*, which has an unusually wide host range). It follows therefore that honey bee viruses will replicate in honey bee-derived cell lines, and potentially in cell lines derived from other hymenopteran species (Table 2). The study of bee viruses in cell culture started with the use of a primary cell line derived from the Asian honey bee (*Apis cerana*) [73]. SBV replicated in this primary cell line, and viral particles were seen by transmission electron microscopy (TEM) after 36 h of infection. The establishment of a continuous honey bee cell line, AmE-711, was reported in 2013 [58] and was used in a single study of virus–virus interactions before the cell line crashed. Honey bees are typically infected by multiple viruses [74] and the AmE-711 cell line was used to examine in vitro competition between viruses in parallel with in vivo experiments [75]. Honey bee virus mixtures were fed to newly emerged honey bees, or used to infect AmE-711 cells with infection dynamics monitored by RT-qPCR [75]. Interestingly, IAPV had a higher replicative advantage among four different viruses (SBV, DWV, IAPV and BQCV), both in vivo and in vitro, even when the virus mixture was predominantly composed of SBV. However, different infection dynamics were observed when KBV was present with a rapid increase in KBV rather than IAPV in cell culture. This work highlights the complexity of virus dynamics within a honey bee with the predominant virus determined in part by the composition of viruses within the honey bee virome at any given time. The results of these in vitro cell culture assays reflected virus dynamics observed in the feeding of live bees, supporting the potential of a honey bee-derived cell line as a powerful tool to study virus infection dynamics. 

Unfortunately, the AmE-711 cell line was persistently infected with DWV, as confirmed by sequence analysis and observation of DWV virions by TEM [75]. While the AmE-711 cell line could have been contaminated during—or subsequent to—establishment, the prevalence of DWV in honey bees and vertical transmission of this virus [76] suggest that DWV was present in the embryos that were used as a starting material. Similarly, previously established primary cell lines as well as the genetically engineered continuous cell line MYN9 were also infected with DWV [47,57]. As vertical transmission of DWV results from virus adherence to the surface of the egg (i.e., transovum transmission) [76], it should be possible to remove the virus from the egg surface using a variety of published procedures [77]. In addition to providing a source of DWV virions, cell lines infected with DWV could be used to assess factors resulting in the switch from a covert to overt DWV infection. For the AmE-711 cell line, the suppressor of RNA interference from *Cricket paralysis virus*, CrPV-1A, was used to induce acute DWV infection and cytopathic effects, confirming the RNAi-mediated suppression of DWV replication in these cells. The AmE-711 cell line was challenging to maintain, likely because environmental stressors (e.g., suboptimal medium, or environmental conditions) weakened the cells allowing DWV titers to increase, similar to the situation in honey bees [78,79]. While the AmE-711 cell line crashed in 2015, it has since been recovered and still harbors DWV. 

## 4. Establishment of Virus-Free Cell Lines 

A variety of continuously replicating cell lines, including vertebrate and invertebrate lines, harbor viruses [80,81,82,83]. Next generation sequencing (NGS) facilitates the discovery of virus-derived sequences in cell lines, and has increased awareness of widespread covert infections in commonly used insect cell lines [84]. Given the widespread occurrence of virus-infected honey bee colonies [85], it is not surprising that virus contamination can be a major problem when establishing *A. mellifera* cell lines. One key example is the AmE-711 cell line, established from *A. mellifera* embryos, which is persistently infected with the DWV [75]. Two studies have described two different approaches for generating virus-free insect cell cultures.

### 4.1. Use of Antiviral Drugs to Establish Virus-Free Insect Cell Lines

A nodavirus, named “ Tn-nodavirus”, was discovered in the BTI-TN-5B1–4 (Tn5) cell line derived from *Trichoplusia ni*, [86] and subsequently in a wide range of *T. ni* cell lines [80]. The IPLB- Sf21 cell line derived from *Spodoptera frugiperda* pupal ovaries, along with the subclonal line, Sf9, are well recognized for generating recombinant proteins via the baculovirus expression system [61]. These Sf cell lines are infected with the Sf-rhabdovirus [81,87]. Maghodia et al. (2017) first treated Sf9 cells with selected anti-viral agents, including ribavirin, 6-azauridune and/or vidarabine, for one month [80]. Although cultures with ribavirin initially appeared to be virus-free, they were later shown to contain the virus when grown in medium without anti-viral drugs. The researchers then isolated single cells using limiting dilution and treated the subclones with antiviral agents. One virus-free clone was generated from this effort [80]. The Sf9-derived, virus-free Sf-RVN cell line is now commercially available (GlycoBac, Laramie, WY). The same drug treatment procedure was repeated to remove the Tn-nodavirus from a *Trichoplusia ni* cell line (Tn-368) with similar results [80]. 

### 4.2. Subcloning to Establish a Virus-Free Cell Line

Ma et al. [81] used limiting dilution to generate virus-free Sf9 subclones in the absence of anti-viral agents from a mixed population of Sf9 cells, comprised of two different virus variants (Sf-rhabdovirus X^+^, X^-^) and uninfected cells. As individual cells failed to survive, a limiting dilution method was used to determine the minimum number of cells required for survival. They transferred 1000 cells/well into one column of a 96-well plate (final volume = 200 µL) and made two-fold serial dilutions into subsequent wells. The wells containing the lowest cell numbers that reached more than 40% confluence after 6–8 weeks were transferred into 24-well plates. A total of 115 cell clones were obtained from fifteen 96-well plates and 18 of these tested as negative for Sf-rhabdovirus. Five of the 18 virus-free clones were further cultured for 30 passages, and three of these clones were confirmed to be virus-free [81]. RNA-seq was used to confirm the absence of reads mapping to the Sf-rhabdovirus genome, for the virus-free cell clone, designated Sf-13F12.

While Sf9 and Tn-368 cells are rapidly replicating cell lines with doubling times of ~24 to 27 h (https://web.expasy.org/cellosaurus), honey bee cell cultures to date have higher doubling times. The AmE-711 cell line, for example, was reported to double every four days [58]. This slow growth rate, combined with cells that are often difficult to culture, suggests that the limiting dilution method will be more challenging for bee cells. To promote cell replication, Reall et al. [68] used a conditioned medium from 72 hr old (log growth phase) parent cell lines, containing naturally produced growth factors, to generate clonal lines from *S. frugiperda* nervous system cell lines (7:3 conditioned medium to fresh medium). Cells were fed every 7 to 10 days with conditioned medium while in the 96-well plate, and with fresh media after they were transferred into T_12.5_ flasks. In ongoing research, we will use a similar procedure to isolate individual cell types from cell cultures that may contain both *A. tumida* and *A. mellifera* cells at BCIRL. Instead of using conditioned medium from potentially virus-containing parental lines, we will generate conditioned medium from actively growing non-bee cell lines (free of bee viruses) and use it to supplement the fresh medium. 

Maghodia et al. [80] mentions additional methods that could be applied for the cloning of *A. mellifera* cell lines, although many of these methods have not been attempted with insect cells. One classic method used to isolate insect cell subpopulations that could be applied to honey bee cells, involves soft agar/agarose overlays followed by colony picking. McIntosh and Rechtoris [88] were the first to use this method on insect cell lines. A more recent modification of this technique uses a feeder layer of actively replicating cells which is overlaid first with 0.2% ultra-pure agarose in 2X medium and then with 0.7% agarose in 2X medium. Low concentrations of well-dispersed cells are then mixed with 0.2% agarose in 2X medium + 72 hr conditioned medium (7:3, as above) to make the final layer [89]. Within a few weeks after the layers are set up, discrete colonies arising from single cells are removed with a pipette. 

Based on the proven approaches described above, it should be feasible to establish virus-free honey bee-derived cell lines in the absence of DWV infection. 

### 4.3. Potential Use of CRISPR/Cas13 for Establishing Virus-Free Cell Lines

An emerging RNA targeting effector Cas13, an RNA-guided single stranded RNA ribonuclease [90], can be employed in conjunction with CRISPR to cleave single strand RNA including both mRNA and the single strand RNA genomes of some RNA viruses. The CRISPR/Cas13 tool has been applied for suppression of viral infections and for virus diagnosis [91]. For suppression of virus infection, CRISPR/Cas13 was transiently expressed in *Nicotiana benthamiana* leaves with guide RNAs (gRNA) targeting multiple regions of the small positive-strand RNA genome of *Turnip mosaic virus* (TuMV; Potyvirus). While gRNAs targeting different regions of the virus genome varied in efficiency, gRNAs targeting HC-pro and GFP sequences resulted in a >50% reduction in virus load [92]. As CRISPR/Cas9 tools have been widely applied in various insect cell lines [93,94], it is conceivable that Cas13 could be employed for suppression of small RNA viruses such as DWV in honey bee-derived cell lines. 

## 5. Potential Applications of Honey Bee Cell Lines

The establishment of virus-free, honey bee cell lines will facilitate a number of avenues of research including (1) screening for antiviral compounds, (2) screening for the potential toxicity of insecticides to honey bees, and (3) elucidation of honey bee-virus molecular interactions. 

### 5.1. Screening of Antiviral Compounds for Use in Apiaries

The cell culture system provides a powerful tool for high-throughput preliminary screening of antiviral drugs [95,96,97] prior to testing of candidate antiviral compounds in the whole organism. This cell line-based screening approach was used to identify candidate compounds for use against the Zika virus [95,96]. While the majority of screens have been conducted in mammalian cell lines, similar strategies could be employed in insect cell culture systems. For example, a high-throughput cell-based screening platform was established to mine compounds for lethality against mosquito cells (*Anopheles* and *Aedes*), but with little or no effect on other insect or human cell lines [98]. This screen resulted in identification of a mosquitocidal compound that had no effect on the vinegar fly, *Drosophila melanogaster*. A honey bee cell line could be employed (1) for the screening of antiviral compounds to reduce viral load within a hive, (2) the screening of current and candidate insecticides for safety to honey bees. The need for such a screening system was highlighted by the impact of neonicotinoid insecticides on honey bee populations [99,100]. 

### 5.2. Elucidation of Molecular Virus–Honey Bee Interactions

A honey bee cell line would allow for in depth study of virus–host molecular interactions. This will be facilitated in particular by the establishment of infectious clones of honey bee viruses such as those of DWV [101,102], that allow for reverse genetic analysis of gene function. Mechanisms of virus binding and entry into the cell, replication, encapsidation and release from the cell along with host cell antiviral response could be delineated by use of a honey bee cell line. A number of virus receptors have been identified from cell culture systems including those for the Epstein–Barr virus (EBV) in human hematopoietic cells [103] and candidate dengue virus (DENV) receptors in mosquito cells [104]. Similarly, the DL2 and S2 cell lines derived from *D. melanogaster* have been used to study the infection cycle, replication of, and RNA interference associated with, small RNA viruses that infect Drosophila [105,106,107]. 

Along with RNAi-, the emerging CRISPR/Cas9 gene editing tool, which has been used in several insect cell lines including Sf9, High Five, BmN [108], S2 [109,110] and Aag2 [111], allows for the identification of host genes involved in viral infection. For example, this system was used to confirm the role of the PIWI-interacting RNA (piRNA) pathway in antiviral response in mosquitoes [112]. A knockout mosquito cell line AF319 was generated by mutating *Dcr2*, a key gene in the RNA interference pathway, using the CRISPR/Cas9 technology. In the *Dcr2* knockout cell line, Piwi4 retained antiviral activity in the absence of the siRNA pathway [111]. The CRISPR/Cas9 gene editing tool also allows for the functional characterization of genes on a genome-wide scale in cell culture systems, and has been used for the discovery of novel drug targets. For example, a CRISPR/Cas9 genome-wide gene knock-out assay in A549 cells was conducted to identify two host factors that are required for *Influenza A virus* (IAV) infection, that could serve as targets for novel antiviral compounds [113]. Similar approaches to these could be adopted for the identification of mechanisms of virus infection, and for antiviral targets for use in the protection of honey bees. 

## 6. Conclusions

Viruses play a significant role in honey bee losses. A honey bee cell line represents a valuable tool to identify solutions to virus infections in apiaries. Previous work with the AmE-711 cell line demonstrated the potential of honey bee cell lines to mirror in vivo virus dynamics. Cell lines derived from hymenopteran species other than *Apis mellifera* may support the replication of some viruses, but would be suboptimal for the study of honey bee-specific viruses. 

Here, we have summarized the establishment of primary and continuous cell lines derived from Hymenoptera. A systematic approach for the establishment of cell lines with the testing of multiple media is warranted for establishment of cell lines from less tractable species such as the honey bee. In addition, methods such as the use of antiviral drugs, sub-cloning and use of CRISPR/Cas13 could be employed for establishment of virus-free, honey bee cell lines. The use of a honey bee cell line in conjunction with virus replicons or infectious clones, and CRIPSR/Cas9-mediated genome editing will facilitate investigation of molecular virus–host interactions. Ultimately, such studies will help mitigate virus-related honey bee losses.

## Figures and Tables

**Table 1:** Primary cell cultures from hymenopteran species.

Species	Tissue	Longevity	Medium	Incubation	Year	Ref
*Pseudomyrmex triplarinus*	Venom glands	1 year	PTM-1CC	28 °C	1985	[26]
*Apis mellifera*	Antennal lobes	~1 month	5+4 and A2	29 °C	1991	[30]
*Apis mellifera*	Pupal honey bee brain	Three weeks	L-15	29 °C	1992	[31]
*Mormoniella vitripennis*	Eggs	3 months	Grace	28 °C	1993	[29]
*Apis mellifera*	Mushroom body	NA	L-15	NA	1994	[32]
*Apis mellifera*	Kenyon cells	Up to 10 days	L-15	29°C	1994	[33]
*Apis mellifera*	Antennal lobe	NA	5+4	NA	1994	[34]
*Apis mellifera*	Antennal flagella	Several weeks	5+4	30 °C	1994	[35]
*Apis mellifera*	Kenyon cells	Up to 6 weeks	L-15	26 °C	1999	[36]
*Apis mellifera*	Antennal motor neurons	NA	L-15	28 °C	1999	[37]
*Apis mellifera*	Kenyon cells and projection neurons	NA	L-15	26 °C	2003	[38]
*Apis mellifera*	Mushroom bodies neuroblasts	NA	L-15	26 °C	2003	[39]
*Apis mellifera*	Antennal lobes	~1 month	L-15	26 °C	2008	[40]
*Apis mellifera*	Pre-gastrulastage embryos	More than 3 months	Grace	30 °C	2006	[44]
*Apis mellifera*	Eggs	Four months	Grace’s or Schneider’s	32 °C with 5% CO2	2010	[45]
*Apis mellifera*	Pupae	At least 8 days	WH2	22 °C	2010	[47]
*Apis mellifera*	Gut	At least 6 days	L-15	33 °C	2012	[46]
*Apis mellifera*	Midgut	15 days	WH2	27 °C	2012	[41]
*Apis mellifera*	Eggs	~135 day	L-15	30 °C	2015	[43]

**Table 2:** Permanent cell lines derived from hymenopteran species.

Species	Stage	Medium	Outcome	Year	Reference
*Neodiprion lecontei*	Embryos	Supplemented Grace’s	10 cell lines	1981	[53]
*Trichogramma pretiosum*	Embryos	IPL-52B + IPL-76 (3:1)	1 cell line	1986	[54]
*Trichogramma confusum*	Embryos	modified IPL-52B	1 cell line	1991	[55]
*Trichogramma exiguum*	Embryos	modified IPL-52B	1 cell line	1991	[55]
*Hyposter didymator*	Pupae	HdM medium	4 cell lines	2004	[56]
*Apis mellifera*	Larvae	Supplemented Grace’s	1 cell line (with c-myc gene)	2011	[57]
*Apis mellifera*	Embryos	HB-1 (modified L-15)	1 cell line	2013	[58]

**Table 3:** Examples of basal media, nutrient supplements and media combinations tested in honey bee cell culture initiations at BCIRL.

Basal Medium ^1^	Supplier	Results ^2^
EX-CELL 420	Millipore Sigma, St Louis, MO	+
TNM-FH	Caisson	+/++
Schneider’s	Caisson	+/++
L-15	Caisson	-
IPL41	Caisson	-
Shields and Sang	Caisson, Smithfield, UT	0/+
DMEM	Millipore Sigma	NT^3^
RPMI-1640	Millipore Sigma	NT
**Medium Supplements**		
9% FBS (heat inactivated)	Millipore Sigma	+++
2% Insect medium supplement (IMS)	Millipore Sigma	-/0/+
1% MEM non-essential amino acids (NEA)	Millipore Sigma	-/0/+
10% Yeast extract	ThermoFisher Scientific, Waltham, MA	+
Royal jelly (RJ)	Made in-house ^4^	++/+++
10 µM 20-hydroxyecdysone	Cayman Chemical, Ann Arbor, MI	0
**Medium Mixtures**	**Reference (If Applicable)**	
HB-1	[58]	+/++
WH5	[47]	+
Kimura’s	[71]	+
EX-CELL 420 + L-15, 1:1 (CLG#2)	[67]	++/+++
TnMFH + IPL41, 1:1 (CLG#4)	N/A	+
Schneider’s + TnMFH + L-15, 1:1:1 (CLG#5)	N/A	+
L-15 + EXCELL 420, 3:1 (HZ#1)	N/A	+
RPMI-1640 + EXCELL 420, 1:1 (HZ#2)	N/A	++/+++
DMEM+EXCELL 420, 1:1 (HZ#3)	N/A	-/0
CLG#2 + RPMI1640 + DMEM, 2:1:1 (HZ#4)	N/A	-/0

^1^All basal media tested contained 9% FBS. ^2^Result key: [–], did not support cell health (vacuoles/granules/dark areas in the cytoplasm and/or no cell attachment and/or cell lysis noted); [0], no visible impact; [+], initially encouraged cell viability and attachment (≤1 month); [++], encouraged cell viability, attachment and replication for > 1 month; [+++], encouraged cell viability and replication such that the culture was passaged at least 1X. Combined scores indicate tissue dependent variability (e.g., -/+, [–] for eggs vs. [+] for queen ovaries and midguts). ^3^ NT = These basal media were only tested in combination with other media +/- supplements. ^4^ Royal jelly was collected fresh from honey bee hives: 100 wax cells were washed off with 0.5 mL CLG#2 and added to 100 mL CLG#2.
